# Efficacy of Active Carbon towards the Absorption of Deoxynivalenol in Pigs

**DOI:** 10.3390/toxins6102998

**Published:** 2014-10-21

**Authors:** Mathias Devreese, Gunther Antonissen, Patrick De Backer, Siska Croubels

**Affiliations:** 1 Department of Pharmacology, Toxicology and Biochemistry, Faculty of Veterinary Medicine, Ghent University, Salisburylaan 133, 9820 Merelbeke, Belgium; E-Mails: gunther.antonissen@ugent.be (G.A.); patrick.debacker@ugent.be (P.D.B.); siska.croubels@ugent.be (S.C.); 2 Department of Pathology, Bacteriology and Avian Diseases, Faculty of Veterinary Medicine, Ghent University, Salisburylaan 133, 9820 Merelbeke, Belgium

**Keywords:** deoxynivalenol, absorption model, pig, mycotoxin binder, efficacy testing

## Abstract

In order to assess the* in vivo* efficacy of mycotoxin binders, specific toxicokinetic parameters should be measured according to European guidelines. For this purpose, an absorption model in pigs is described with emphasis on absorption kinetics. Pigs received a single oral bolus of the mycotoxin deoxynivalenol alone or in combination with active carbon (applied as mycotoxin binder). After administration of deoxynivalenol alone, significant plasma amounts of deoxynivalenol were detected and kinetic parameters were calculated using a one compartmental model. Activated carbon completely prevented the absorption of deoxynivalenol as no plasma amounts could be detected.

## 1. Introduction

The contamination of feed with mycotoxins is a continuing feed safety issue leading to economic losses in animal production [1]. Consequently, a variety of methods for the decontamination of feed have been developed, but mycotoxin detoxifying agents seem to be the most promising and are therefore most commonly used [2,3]. These detoxifying agents can be divided into two different classes, namely mycotoxin binders and mycotoxin modifiers. These two classes have different modes of action; mycotoxin binders adsorb the toxin in the gut, resulting in the excretion of complex toxin-binder in the faeces, whereas mycotoxin modifiers transform the toxin into non-toxic metabolites [4]. The extensive use of these additives led, in 2009 in the European Union, to the establishment of a new group of feed additives called mycotoxin detoxifiers. These compounds are specified as “substances for reduction of the contamination of feed by mycotoxins: substances that can suppress or reduce the absorption, promote the excretion of mycotoxins or modify their mode of action” [5].

The efficacy of these products has to be evaluated. *In vivo* efficacy trials are usually based on so-called unspecific parameters, evaluating animal performance, blood biochemical or hematological parameters, organ weight, effects on immune function, histological changes,* etc.* [6]. As these criteria are non-specific, differences obtained between treated and untreated animals cannot be solely attributed to the efficacy of the detoxifier. There may be confounding effects involved such as immuno-modulating activity of β-glucans and antioxidant action of other feed components. A possibility to distinguish between specific and unspecific effects is the inclusion of a group fed non-contaminated feed supplemented with the detoxifier. However, the European Food Safety Authority (EFSA) proposed other end-points based on specific toxicokinetic parameters [7]. As mycotoxin binders are deemed to adsorb mycotoxins in the gut, a lowered intestinal absorption is expected. According to the EFSA, the most relevant parameter to evaluate the efficacy of these products is the plasma concentration of these toxins or their main metabolites. The EFSA proposes short-term feeding trials where the mycotoxin and detoxifier is mixed in the feed [7]. The plasma concentrations of the mycotoxin, and the main metabolite(s), should be monitored over a period of at least 5 days with a presampling period of at least one week (steady-state design). Furthermore, unspecific parameters may be monitored as well. Such feeding trials are labor and cost intensive. In contrast, a toxicokinetic model where the mycotoxin is administered with or without mycotoxin detoxifier as a single oral administration would be less expensive and labor intensive to perform.

The aim of present study was to evaluate a bolus absorption model in relation to the EFSA guidelines, to study the efficacy of mycotoxin detoxifiers towards the oral absorption of deoxynivalenol (DON) in pigs.

## 2. Results and Discussion

**Figure 1:**
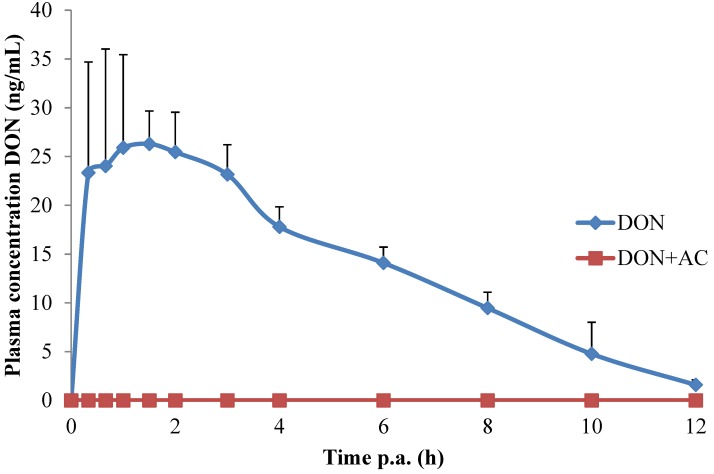
Plasma concentration-time profile of deoxynivalenol in piglets after single oral bolus administration of deoxynivalenol (DON) (0.05 mg/kg bw) alone (DON, *n* = 4) or in combination with activated carbon (DON + AC, *n* = 4). No plasma concentrations of DON were detected above the limit of detection (0.05 ng/mL) in the DON + AC group. Values are presented as mean + SD.

After single oral bolus administration of 0.05 mg DON/kg bw, quantifiable plasma amounts of DON were detected (Figure 1). No statistical differences in absorption parameters between males and females were found (data not shown). The plasma concentration-time profile fitted a one compartmental model. The *T_max_* of 1.33 h is comparable to the value of 1.65 h reported by Goyaerts and Dänicke (2006) [8]. The *C_max_* of 29.7 ng/mL on the other hand, was higher compared to [8] (15.1 ng/mL after oral dosing of 0.08 mg DON/kg bw). However, feed intake can influence the oral bioavailability of DON which explains the slightly higher *C_max_* in the present study with fasted pigs in comparison to fed pigs as used in [8]. Other kinetic parameters of DON (Table 1) were comparable to literature reports [8,9]. The major metabolite of DON, de-epoxydeoxynivalenol (DOM-1), was not detected in plasma in the present study. This correlates to previous literature reports where DOM-1 only accounted for 1.4%–1.7% of the total DON concentration in the systemic circulation of pigs [10].

To test the effectiveness of this model in pigs, DON was also administered in combination with active carbon (AC) as it was demonstrated that it strongly adsorbs DON in broiler chickens [11]. The absorption of DON was completely prevented by AC as no DON, above the limit of detection (LOD), could be detected in plasma. This demonstrates the suitability of the absorption kinetic model to evaluate the efficacy of mycotoxin binders towards the oral absorption of DON in pigs. As stated, AC was used as a positive control because it is a universal antidote which adsorbs various compounds, including mycotoxins such as DON [12,13]. However, the commercial use of AC in practice should be avoided in order to minimize the risk of a diminished nutrient absorption as well as the impairment of nutritional value of the feed [12].

**Table 1:** Main absorption kinetic parameters of deoxynivalenol (DON) after single oral administration of DON to piglets (0.05 mg/kg bw, *n* = 4). Values are presented as mean ± SD.

Kinetic parameter	DON
*C_max_* (ng/mL)	29.73 ± 6.847
*T_max_* (h)	1.33 ± 0.802
*AUC_0-inf_* (ng.h/mL)	168.62 ± 22.682
k_a_ (h^−1^)	3.87 ± 2.95
*T_1/2a_* (h)	0.66 ± 0.650
k_el_ (h^−1^)	0.26 ± 0.048
*T_1/2el_* (h)	2.73 ± 0.477
*Vd/F* (L/kg)	1.19 ± 0.024
*Cl/F* (L/h/kg)	0.30 ± 0.042

*C_max_* = maximal plasma concentration; *T_max_* = time to maximal plasma concentration; *AUC_0-inf_* = area under the plasma concentration-time curve from time 0 to infinite; k_a_ = absorption rate constant; *T_1/2a_* = absorption half-life; k_el_ = elimination rate constant; *T_1/2el_* = elimination half-life; volume of distribution divided by the oral bioavailability = *Vd/F* and clearance divided by the oral bioavailability =* Cl/F*.

## 3. Experimental Section

### 3.1. Chemicals, Products and Reagents

The standard of DON, used for the animal and analytical experiments, was obtained from Fermentek (Jerusalem, Israel). DOM-1 was purchased from Sigma-Aldrich (Bornem, Belgium). Internal standard (IS), ^13^C_15_-DON, was purchased from Biopure (Tulln, Austria). The standards were stored at ≤−15 °C. Water, methanol and acetonitrile (ACN) were of LC-MS grade and were obtained from Biosolve (Valkenswaard, The Netherlands). Glacial acetic acid was of analytical grade and obtained from VWR (Leuven, Belgium). Millex^®^-GV-PVDF filter units (0.22 µm) were obtained from Merck-Millipore (Diegem, Belgium).

### 3.2. Animal Experiment

Eight piglets (20.2 ± 1.4 kg bw) of mixed gender were purchased (Biocentre Agrivet, Merelbeke, Belgium) and housed in four different compartments (±4 m^2^/compartment, two animals/compartment). The temperature was kept between 18 and 25 °C. The relative humidity was between 40% and 80%. An ambient day-light scheme was applied. After a one week acclimatization period, the pigs were fasted for 12 h followed by administration of a single oral bolus of 0.05 mg DON/kg bw by oral gavage using an intragastric tube. This dose resembles a feed contamination amount of 1 mg DON/kg. For this bolus administration, DON was dissolved in ethanol (1 mg/mL) and further diluted with tap water up to a volume of 10 mL. Four of the eight pigs received the DON bolus in combination with activated carbon (AC) (0.1 g/kg bw, resembling an inclusion amount of 2 g/kg feed) (NORIT Carbomix^®^, KELA Pharma, Sint-Niklaas, Belgium), suspended in 10 mL of tap water. Immediately after administration of the bolus, the intragastric tube was rinsed with 50 mL of tap water. Blood samples were drawn before (0 min) and at 0.33, 0.66, 1, 1.5, 2, 3, 4, 6, 8, 10 and 12 h post administration. Blood samples were taken in heparinized tubes and centrifugated (2851 × *g*, 10 min, 4 °C). Aliquots (250 µL) of plasma samples were stored at ≤−15 °C until analysis. This animal experiment was approved by the Ethical Committee of Ghent University (Case number EC 2011-13).

### 3.3. Quantification of DON in Plasma

Samples were analyzed as previously described by Devreese* et al.* (2012) [14]. Briefly, 12.5 µL of IS and 750 µL of ACN were added to 250 µL of plasma, followed by vortex mixing (15 s) and centrifugation (8517 × *g*, 10 min, 4 °C). Next, the supernatant was transferred to another tube and evaporated using a gentle nitrogen (N_2_) stream (45 ± 5 °C). The dry residue was reconstituted in 200 µL of water/methanol (85/15, *v*/*v*). After vortex mixing (15 s), the sample was passed through a Millex^®^ GV-PVDF filter (0.22 µm) and transferred into an autosampler vial. An aliquot (5 µL) was injected onto the LC-MS/MS instrument. The LC system consisted of a quaternary, low-pressure mixing pump with vacuum degassing, type Surveyor MSpump Plus and an autosampler with temperature controlled tray and column oven, type Autosampler Plus, from ThermoFisher Scientific (Breda, The Netherlands). Chromatographic separation was achieved on a Hypersil^®^ Gold column (50 mm × 2.1 mm internal diameter, particle diameter: 1.9 µm) in combination with a guard column of the same type (10 mm × 2.1 mm internal diameter, particle diamter: 3 µm), both from ThermoFisher Scientific. A gradient elution program was performed with 0.1% glacial acetic acid in water and methanol as mobile phases. The LC column effluent was interfaced to a TSQ^®^ Quantum Ultra triple quadrupole mass spectrometer, equipped with a heated electrospray ionization (h-ESI) probe operating in the negative ionization mode (all from ThermoFisher Scientific). Following selected reaction monitoring (SRM) transitions were monitored and used for identification and quantification, respectively: for DON *m*/*z* 355.1 > 265.2 and 355.1 > 295.1, for DOM-1 *m*/*z* 339.1 > 459.1 and 339.1 > 249.0 and for ^13^C_15_-DON *m*/*z* 370.1 > 279.2 and 370.1 > 310.1. The limit of quantification (LOQ) of DON and DOM-1 was 1 and 2 ng/mL, respectively, whereas the limit of detection (LOD) was 0.05 and 0.04 ng/mL, respectively.

### 3.4. Absorption Parameters

Parameter analysis was performed with WinNonlin 6.3. (Pharsight, St. Louis, MO, USA). The most important parameters of DON were calculated: maximal plasma concentration (*C_max_*), time to maximal plasma concentration (*T_max_*), area under the plasma concentration-time curve from time 0 to infinite (*AUC_0-inf_*), absorption rate constant (k_a_), absorption half-life (*T_1/2a_*), elimination rate constant (k_el_), elimination half-life (*T_1/2el_*), volume of distribution divided by the oral bioavailability (*Vd/F*) and clearance divided by the oral bioavailability (*Cl/F*).

## 4. Conclusions

For the first time, an* in vivo* model was applied to evaluate the efficacy of active carbon towards the oral absorption of deoxynivalenol in pigs, based on absorption kinetic characteristics. Activated carbon completely prevented the absorption of DON from the intestinal tract.
